# Dysfunction of miR‐802 in tumors

**DOI:** 10.1002/jcla.23989

**Published:** 2021-09-24

**Authors:** Tong Gao, Mengsha Zou, Tiancheng Shen, Shiwei Duan

**Affiliations:** ^1^ Medical Genetics Center Ningbo University School of Medicine Ningbo China; ^2^ School of Medicine Zhejiang University City College Hangzhou China

**Keywords:** ceRNA, diagnosis, miR‐802, signaling pathway, target gene, tumor

## Abstract

Recent studies have shown that miR‐802 is abnormally expressed in many tumors. miR‐802 is expressed at low levels in tissues and cells of gastric cancer, colorectal cancer, breast cancer, cervical cancer, epithelial ovarian cancer, tongue squamous cell carcinoma, oral squamous cell carcinoma, esophageal squamous cell carcinoma, laryngeal squamous cell carcinoma, and melanoma. In contrast, miR‐802 is overexpressed in hepatocellular carcinoma, bladder urothelial cancer, osteosarcoma, and cholesteatoma tissue cells. It should be noted that the results of studies on the expression of miR‐802 in pancreatic cancer, prostate cancer, and lung cancer are inconsistent. Current studies have found that miR‐802 can target and regulate genes in different tumors, and affect the regulation of the Wnt signaling pathway, EMT signaling pathway, PI3K/AKT signaling pathway, ERK signaling pathway, and Hedgehog signaling pathway. At the same time, miR‐802 is regulated by the endogenous competition of four ceRNAs, including circDONSON, IGFL2‐AS1, MIR155HG, and MIR4435‐2HG. This article reviews the abnormal expression of miR‐802 in a variety of tumors, expounds the mechanism by which miR‐802 affects tumor progression by regulating different target genes, and elaborates the network of miR‐802‐related ceRNAs. We also summarized the limitations of miR‐802 research and looked forward to the potential application of miR‐802 in the diagnosis and prognosis of tumors.

## Graphical abstract

Current studies have found that miR‐802 is abnormally expressed in a variety of tumor tissues and cells, suggesting that this review of miR‐802 summarizes the abnormal expression of miR‐802 in a variety of tumor diseases and analyzes how miR‐802 regulates differently.

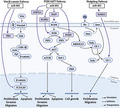

## 1 INTRODUCTION

MicroRNAs (miRNAs) are a class of small ribonucleic acids (19–24 nucleotides).^1^ miRNAs play an important role in post‐transcriptional gene silencing (PTGS).^1^ Thousands of miRNAs have been discovered, and they can regulate more than 30% of gene expression.^2^ The miRNAs can be detected in about 50% of the mutated regions in tumor tissues, suggesting that miRNAs may be closely related to the occurrence and development of tumors.^2^

MicroRNA‐802 (miR‐802) gene is located on chromosome 21, and its precursor can be processed into two functional mature miRNAs (miR‐802‐3p and miR‐802‐5p). Among them, miR‐802‐3p is much richer than miR‐802‐5p.^3^ Current studies have found that miR‐802 is abnormally expressed in a variety of tumor tissues and cells, suggesting that the expression of miR‐802 may be related to tumors. This review reports the abnormal expression of miR‐802 in various tumors including tongue squamous cell carcinoma (TSCC), oral squamous cell carcinoma (OSCC), esophageal squamous cell carcinoma (ESCC), laryngeal squamous cell carcinoma (LSCC), gastric cancer (GC), colorectal cancer (CRC), pancreatic cancer (PC), hepatocellular carcinoma (HCC), prostate cancer (PCa), bladder urothelial cancer, breast cancer (BC), cervical cancer (CC), epithelial ovarian cancer (EOC), lung cancer, osteosarcoma, cholesteatoma, and melanoma. We also summarized the diagnostic and prognostic value of miR‐802 in tumors. We proposed the mechanism by which miR‐802 affects the occurrence and development of these tumors. In addition, we summarized the role of circDONSON, lncRNA IGFL2‐AS1, MIR155HG, and MIR4435‐2HG in the regulation of miR‐802, and prospected the application value of miR‐802 in tumors.

## 2 ABERRANT EXPRESSION OF MIR‐802 IN TUMORS

As shown in Table 1, miR‐802 is abnormally expressed in most tumors. As a tumor suppressor gene, miR‐802 can inhibit the occurrence and development of tumors. Some studies have also confirmed the tumor suppressor effect of miR‐802 in mouse models. Existing studies have found that the expression of miR‐802 is decreased in at least 10 cancers, including TSCC,^4^ OSCC,^5^, ^6^ ESCC,^7^ LSCC,^8^ GC,^9^, ^10^, ^11^ CRC,^12^, ^13^, ^14^ BC,^15^, ^16^ CC,^17^, ^18^, ^19^ EOC,^20^ and melanoma.^21^ It is worth noting that the direction of abnormal expression of miR‐802 in the other 4 tumors is inconsistent. In HCC,^22^, ^23^, ^24^, ^25^ bladder urothelial carcinoma,^26^ osteosarcoma,^27^, ^28^ and cholesteatoma,^29^ the expression level of miR‐802 is elevated. This indicates that miR‐802 can also promote the occurrence and development of tumors.

**TABLE 1:** The role of miR‐802 in different human cancers

Tumor type	Expression level	Number of clinical samples	Assessed cell lines	Effect in vitro	Effect in vivo	Regulatory mechanism	References
TSCC	Down‐regulated	12 tumor samples and 9 normal samples	SCC1, SCC4, Cal27, UM1, NHOK, NOK16B	Cell viability↓, invasion↓	–	–	4
OSCC	Down‐regulated	23 paired tissues	–	–	–	–	5
	Down‐regulated	50 paired tissues	Tca8113, SCC9, SCC25, CAL27, HN12, HSU3, FADU	Cell viability↓, colony formation↓, migration and invasion↓	Tumor growth↓	–	6
ESCC	Down‐regulated	120 paired tissues		Proliferation↓, migration↓	–	–	7
LSCC	Down‐regulated	Normal tissue and postoperative laryngeal carcinoma	HEp‐2, SNU899, TU212, HEK293T, BEAS‐2B	Cell colony formation↓, proliferation↓, apoptosis↑	–	–	8
GC	Down‐regulated	46 paired tissues	SGC‐7901, MGC‐803, HGC‐27, BGC‐823	Proliferation↓, Colony Formation↓, Apoptosis↑	Tumor growth↓	–	9
	Down‐regulated	60 paired tissues	AGS, HGC‐27	Cell viability↓, DDP resistance↓, apoptosis↑	–	CircRNA DONSON/miR‐802 / BMI1	10
	Down‐regulated	40 paired tissues	SGC‐7901, BGC‐823, AGS	Proliferation↓, migration and invasion↓	Tumor growth↓	lincRNA IGFL2‐AS1 / miR‐802 / ARPP19	11
CRC	Down‐regulated	38 paired tissues	HCT‐116, LS174T, SW480, NCM460	Migration and invasion↓, cell viability↓	–	–	12
	Down‐regulated	45 primary CRC tissues, 34 paired adjacent non‐tumor tissues, 51 metastatic tissues	SW480, HT29, HCT‐8, SW620, HCT116, DLD‐1, NCM460	Proliferation↓, migration and invasion↓, apoptosis↑	Tumor growth↓	miR‐802/Ran/EGFR,d activating ERK; AKT signaling pathway	13
	Down‐regulated	75 paired tissues	HCT116, HCT8, HT29, DLD‐1, NCM460	Proliferation↓, migration and invasion↓, apoptosis↑	Tumor growth↓	miR‐802/ UBN2	14
PC	Down‐regulated	6 tumor samples and 5 normal samples	MiaPaCa	–	–	Wnt pathway	30
	Down‐regulated	38 paired tissues	AsPC‐1, BxPC‐3, PANC‐1, SW1990,HPDE6‐C7	Cell cycle arrest↑, apoptosis↑, proliferation↓	–	MIR155HG/miR‐802	31
	Up‐regulated	Samples of cyst fluid from IPMN, mucinous cystic neoplasms and pancreatic cancer patients	–	–	–	–	32
HCC	Up‐regulated	172 tumor blood samples and 60 normal blood samples; 48 paired samples	SMMC7721, MHCC97, Bel‐7402, Huh7, Hep3B, HL‐7702, THLE‐3, HEK 293T	T‐cell viability↓	–	–	22
	Up‐regulated	30 paired tissues	Human HCC cell line HepG2.2.15 with the HBV infection and HCC cell line HepG2 without HBV infection	HBV expression and replication↓	–	–	23
	Up‐regulated	Normal tissue and HCC carcinoma	Hep3B, Huh7, SMMC‐7721, Bel‐7402, LO2	Proliferation↑, cell viability↑, cell colony formation↑	Tumor growth↑	AKT phosphorylation signaling pathway	24
	Up‐regulated	12 paired tissues	SMMU‐7721	Proliferation↑, migration↑, cell cycle progression↑	Tumor growth↑	–	49
PCa	Up‐regulated	60 paired tissues	‐	Proliferation↑, migration↑	–	Hedgehog(Hh)signaling pathway	2
	Down‐regulated	73 paired tissues	PC3, DU145, RWPE‐1	Proliferation↓, migration and invasion↓, apoptosis↑	Tumor growth↓	EMT pathway	33
Bladder Urothelial Cancer	Up‐regulated	127 paired tissues	T24, 5637, J82, BIU‐87, SW870, UM‐CM‐3, SV‐HUC‐1	Proliferation↑, migration↑	–	EMT pathway	26
BC	Down‐regulated	20 paired tissues	Breast cancer cell lines (MCF‐7, MDA‐MB‐453, MDA‐MB‐468 and ZR‐75‐1) and normal breast epithelial cells (HBL‐100)	Proliferation↓	Tumor growth↓	miR‐802/FoxM1	15
	Down‐regulated	4 tumor samples	Murine mammary carcinoma 4T1 cell line	–	–	–	16
CC	Down‐regulated	40 paired tissues	Ect1, E6E7, HeLa, C‐33 A, SiHa, ME‐180	Migration and invasion↓, cell viability↓	–	EMT pathway	17
	Down‐regulated	38 paired tissues	HaCaT and four human cervical cancer cell lines	Proliferation↓, cell colony formation↓, apoptosis↑	–	–	18
	Down‐regulated	–	–	Growth↓, invasion↓	Tumor growth↓	miR‐802/MYLIP	19
EOC	Down‐regulated	35 paired tissues	OVCAR3, SKOV3, A2780, CAOV3	Invasion and migration↓, proliferation↑, apoptosis↑	–	–	20
Lung cancer	Down‐regulated^a^	52 paired tissues	A549, H522, H1299, H460, SK‐MES‐1, BEAS‐2B	Proliferation↓, cell colony formation↓, migration and invasion↓, apoptosis↑	Tumor growth↓	PI3K/Akt/Mtor signaling pathway	34
	Up‐regulated	40 paired tissues	A549, NCI‐H358, NCI‐H1299	Proliferation↑	–	miR‐802/Menin; Wnt/β‐catenin; NF‐κB/p65 signaling pathway	35
	Down‐regulated	–	A549	Migration and invasion↓	–	–	36
Osteosarcoma	Up‐regulated	10 tumor samples and 2 normal samples	–	–	–	–	27
	Up‐regulated	–	U2OS, MG63	Proliferation↑	–	–	28
Cholesteatoma	Up‐regulated	11 paired tissues	‐	Proliferation↑	–	NF‐κb/miR‐802/PTEN signaling pathway	29
Melanoma	Down‐regulated	28paired tissues	A375, A2058	Proliferation↓, migration and invasion↓	–	MIR4435‐2HG/miR‐802/FLOT2	21

Abbreviations: ↑, promotion; ↓, inhibition; BC, breast cancer; CC, cervical cancer; CRC, colorectal cancer; DDP, cisplatin; EMT, epithelial‐mesenchymal transition; EOC, epithelial ovarian cancer; ESCC, esophageal squamous cell carcinoma; GC, gastric cancer; HCC, hepatocellular carcinoma; LSCC, laryngeal squamous cell carcinoma; OSCC, oral squamous cell carcinoma; PC, pancreatic cancer; PCa, prostate cancer; TSCC, tongue squamous cell carcinoma; Wnt, wingless/integrated.

^a^ Non‐small cell lung cancer.

In addition, in PC,^30^, ^31^, ^32^ PCa,^2^, ^33^ and lung cancer,^34^, ^35^, ^36^ the results of the association between miR‐802 expression levels and cancer risk are contradictory. In PC, two studies have reported low expression of miR‐802^30^, ^31^ in tumor tissues and cells. However, the expression level of miR‐802 in highly aggressive (HG‐I) PC cyst fluid was found to be 131 times that of low‐grade benign (LG‐B) PC cyst fluid.^32^ In PCa, miR‐802 expression in PCa tissue samples and prostate cells was significantly reduced, and the tumor suppressor effect of miR‐802 was further verified by both in vivo and in vitro experiments.^33^ However, miR‐802 expression in PCa tissue was found to be higher than that in adjacent non‐tumor tissues.^2^ In lung cancer, two studies have found that miR‐802 is expressed at low levels in cancer tissues and cells. Both in vivo and in vitro experiments have further verified the tumor suppressor effect of miR‐802.^34^, ^36^ However, miR‐802 expression in lung cancer tissues was significantly up‐regulated. In vitro experiments also further showed that miR‐802 promoted the proliferation of lung cancer cells.^35^

The abnormal expression of miR‐802 has been confirmed in the tumor tissues or tumor cells of 15 cancers, including TSCC,^4^ OSCC,^5^, ^6^ LSCC,^8^ GC,^9^, ^10^, ^11^ CRC,^12^, ^13^, ^14^ PC,^30^, ^31^, ^32^ HCC,^22^, ^23^, ^24^, ^25^ PCa,^2^, ^33^ bladder urothelial cancer,^26^ BC,^15^, ^16^ CC,^17^, ^18^, ^19^ EOC,^20^ lung cancer,^34^, ^35^, ^36^ osteosarcoma,^27^, ^28^ and melanoma.^21^ To note, the abnormal expression of miR‐802 in ESCC^7^ and cholesteatoma^29^ has only been confirmed in tumor cells.

Subsequent experiments further revealed that miR‐802 expression can regulate a series of cell behaviors, including cell proliferation,^2^, ^7^, ^8^, ^9^, ^11^, ^13^, ^14^, ^15^, ^18^, ^20^, ^21^, ^22^, ^24^, ^25^, ^26^, ^28^, ^29^, ^31^, ^33^, ^34^, ^35^ invasion,^4^, ^6^, ^11^, ^12^, ^13^, ^14^, ^17^, ^19^, ^20^, ^21^, ^33^, ^34^, ^36^ metastasis,^2^, ^6^, ^7^, ^11^, ^12^, ^13^, ^14^, ^17^, ^20^, ^21^, ^25^, ^26^, ^33^, ^34^, ^36^ apoptosis,^8^, ^9^, ^10^, ^13^, ^18^, ^20^, ^31^, ^33^, ^34^ colony formation,^6^, ^8^, ^9^, ^18^, ^24^, ^34^ viability,^4^, ^6^, ^10^, ^12^, ^17^, ^22^, ^24^ cell cycle arrest,^25^, ^31^ and cisplatin resistance.^10^

In addition, some studies have confirmed the inhibitory effect of miR‐802 on tumor growth, including OSCC,^6^ GC,^9^, ^11^ CRC,^13^, ^14^ PCa,^33^ BC,^15^ CC,^19^ and lung cancer.^34^ However, other studies have confirmed the promotion of miR‐802 on the tumor growth of HCC.^24^, ^25^

## 3 PROGNOSTIC VALUE OF ABNORMAL EXPRESSION OF MIR‐802

As shown in Table 2, our study found that the expression level of miR‐802 is related to the prognosis of patients with tumors. miR‐802 expression is found to be reduced in ESCC, CRC, and EOC (Table 2). In ESCC, high expression of miR‐802 is significantly associated with the increase in overall survival (OS) and progression‐free survival (PFS).^7^ In EOC, highly expressed miR‐802 can be used as an independent prognostic factor and is significantly related to the increase in PFS and time to progression (TTP).^37^ In CRC, low expression of miR‐802 is an independent prognostic factor affecting OS in patients, and decreased expression of miR‐802 is associated with the progression of CRC and poor prognosis of patients.^14^ In contrast, high expression of miR‐802 was significantly associated with poor OS and cancer‐specific survival (CSS) of CRC.^38^

**TABLE 2:** Diagnostic or prognostic value of miR‐802 expression

Cancer type	Sample size	Expression pattern	Diagnostic/prognostic value	References
ESCC	120 paired tissues	Down‐regulated	Prognostic factor of OS and PFS	36
EOC	197 tumor samples	Down‐regulated	Independent predictors of PFS and TTP	37
CRC	147 tumor samples	Down‐regulated	Prognostic factor of OS and CSS	38
	75 paired tissues	Down‐regulated	Independent predictors of OS	13
HCC	172 tumor blood samples and 60 normal blood samples; 48 paired samples	Up‐regulated	Prognostic factor of OS	21
	Normal tissue and HCC carcinoma	Up‐regulated	Prognostic factor of OS and DFS	23
PCa	60 paired tissues	Up‐regulated	Prognostic factor of OS	2

Abbreviations: CRC, colorectal cancer; CSS, cancer‐specific survival; DFS, disease‐free survival; EOC, epithelial ovarian cancer; ESCC, esophageal squamous cell carcinoma; HCC, hepatocellular carcinoma; OS, overall survival; PCa, prostate cancer; PFS, progression‐free survival; TTP, time to progress.

However, miR‐802 expression is elevated in HCC and PCa. The OS and disease‐free survival (DFS) of HCC patients with high expression of miR‐802 are shorter.^24^ Jiang et al. further found that the median survival time of patients with high blood miR‐802 levels was much shorter than that of patients with low blood miR‐802 levels.^22^ Low blood miR‐802 expression is significantly related to the good prognosis of HCC patients, but the expression level of miR‐802 in tissues is not significantly correlated with the prognosis of HCC patients.^22^ In PCa, the 3‐year survival rate and OS of the miR‐802‐negative group were significantly higher than those of the miR‐802‐positive group.^2^

## 4 MIR‐802 AFFECTS TUMOR PROGRESSION BY REGULATING THE EXPRESSION OF TARGET GENES

**FIGURE 1:**
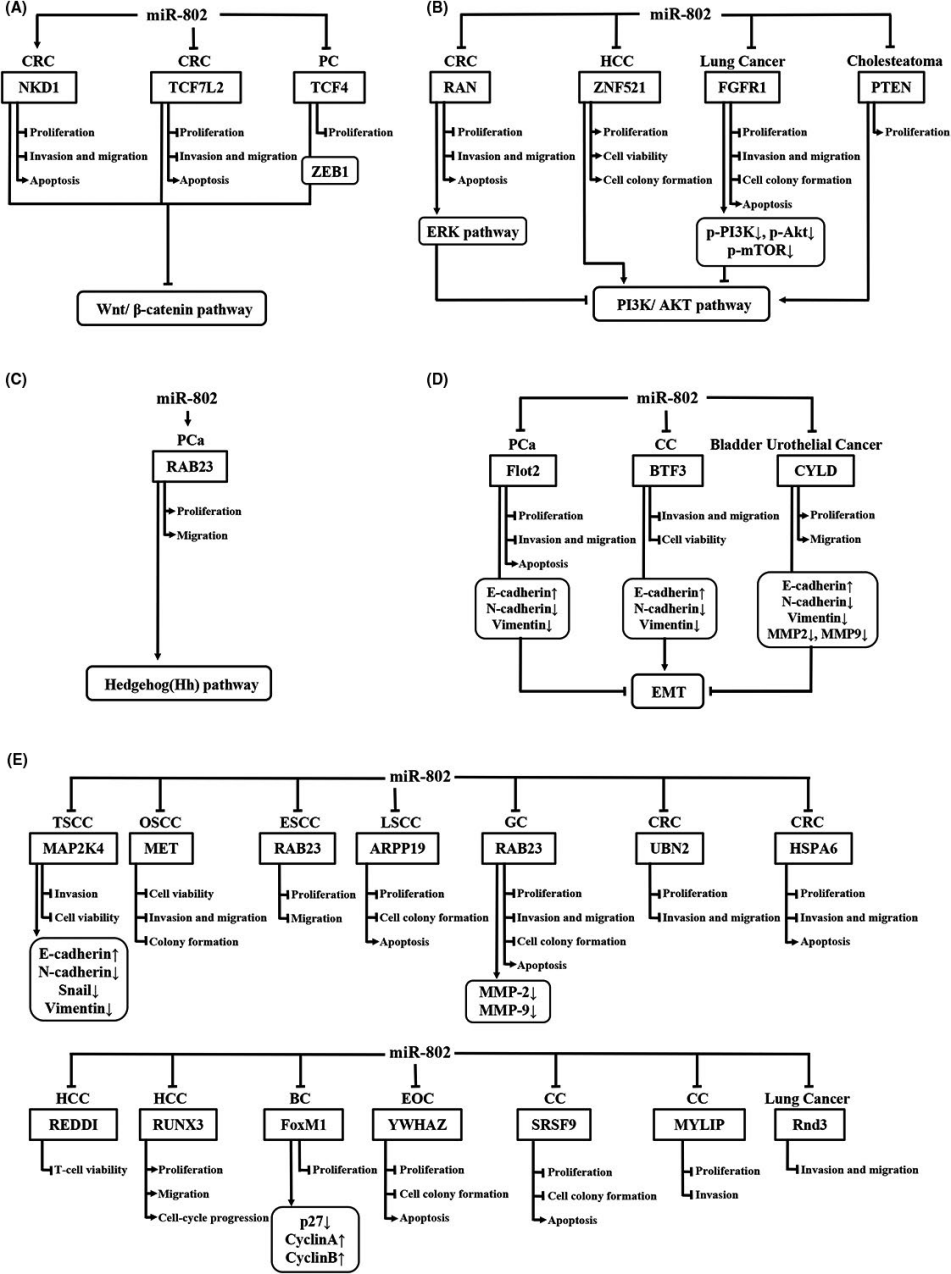
Potential downstream regulatory mechanisms of miR‐802. MiR‐802 influences downstream targets as well as (A) the Wnt/β‐catenin pathway, (B) the PI3K/AKT signaling pathway, (C) Hedgehog (Hh) pathway, (D) the EMT signaling pathways, and (E) other mechanisms, thereby affecting proliferation, invasion, metastasis, and apoptosis of tumor cells. ↑, promotion; ↓, inhibition; BC, breast cancer; CC, cervical cancer; CRC, colorectal cancer; CRC, colorectal cancer; EMT, epithelial‐mesenchymal transition; EOC, epithelial ovarian cancer; ESCC, esophageal squamous cell carcinoma; HCC, hepatocellular carcinoma; LSCC, Laryngeal squamous cell carcinoma; MMP‐2, Matrix metalloproteinase 2; MMP‐9, Matrix metalloproteinase 9; OSCC, oral squamous cell carcinoma; PC, pancreatic cancer; PCa, prostate cancer; PI3K/ AKT, phosphatidylinositol‐3‐kinase/protein kinase B; TSCC, tongue squamous cell carcinoma; Wnt: Wingless/ Integrated

**FIGURE 2:**
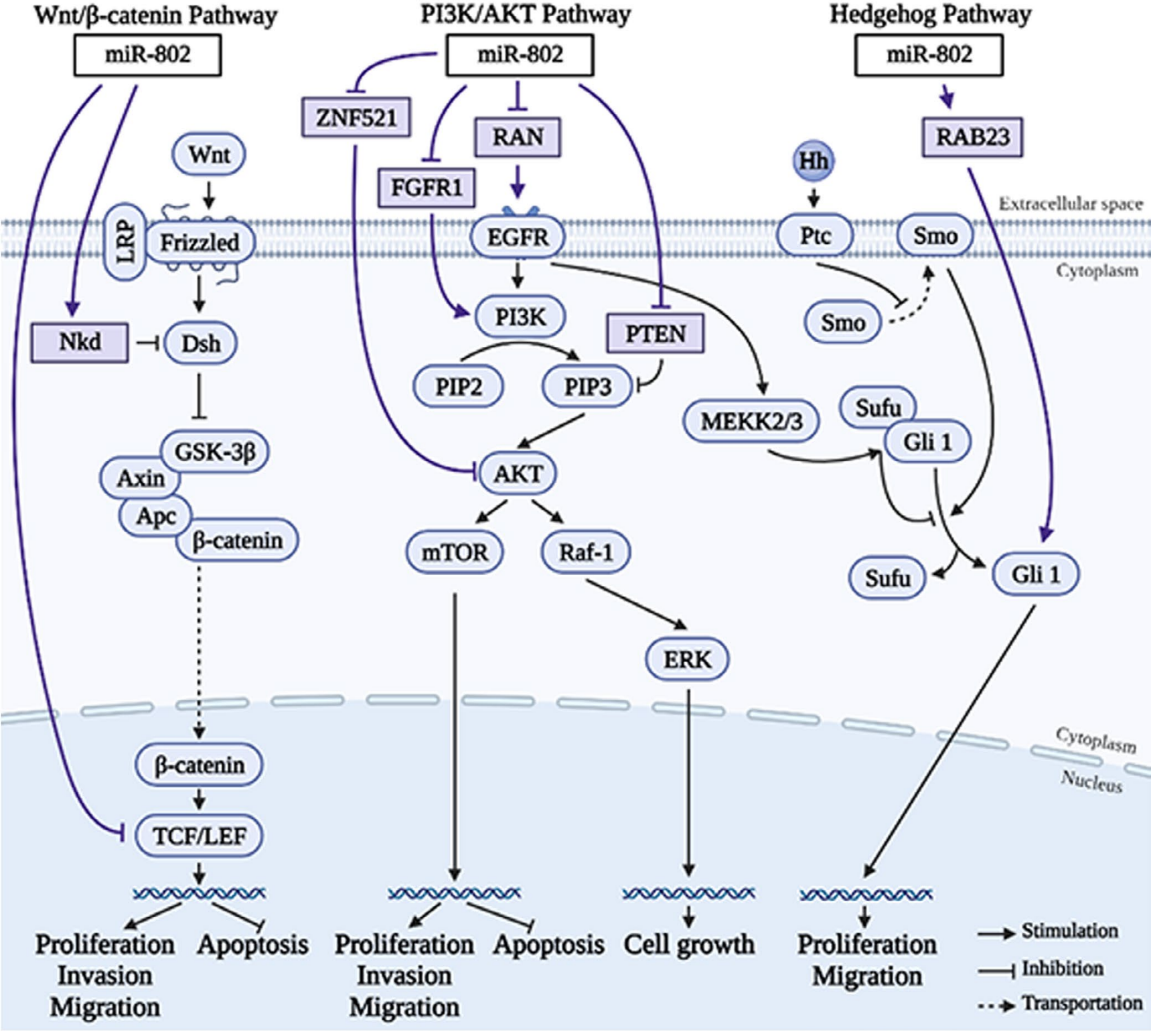
miR‐802 regulates the biological processes of cells by affecting signaling pathways. miR‐802 regulates the signal molecules in the Wnt/β‐catenin signaling pathway, the PI3K/AKT signaling pathway, Hedgehog (Hh) signaling pathway

As shown in Figures 1 and 2, miR‐802 regulates the expression and protein activity of a series of downstream genes through the miR‐802/mRNA axis, thereby affecting tumor progression. In 17 types of tumors, miR‐802 targets at least 22 genes, including *NKD1*, *TCF7L2*, *TCF4*, *Menin*, *Flot2*, *CYLD*, *BTF3*, *RAN*, *UBN2*, *ZNF521*, *FGFR1*, *HSPA6*, *RAB23*, *MAP2K4*, *MET*, *ARPP19*, *REDDI*, *FoxM1*, *YWHAZ*, *SRSF9*, *Rnd3*, and *MYLIP*. miR‐802 affects the activation of the Wnt signaling pathway, EMT signaling pathway, PI3K/AKT signaling pathway, ERK signaling pathway, and Hedgehog signaling pathway. Thus, miR‐802 plays an important role in cell behaviors, including tumor cell proliferation, invasion, metastasis, and apoptosis.

### 4.1 miR‐802 and the Wnt signaling pathway

As one of the most classic signaling pathways, the Wnt signaling pathway plays a key role in the occurrence and development of many cancers.^39^ NKD1 was found to be an evolutionary conservative feedback inhibitor of the Wnt signaling pathway.^40^ TCF7L2 is an important part of the Wnt/β‐catenin signaling pathway.^41^ TCF4 encodes a transcription factor in the Wnt signaling pathway.^30^ As shown in Figures 1A and 2, overexpressed miR‐802 can up‐regulate the expression of its target gene NKD1, thereby inhibiting the proliferation, migration, invasion of CRC cells, and promoting apoptosis of CRC cells.^42^ Meanwhile, overexpressed miR‐802 exerts a tumor suppressor effect by down‐regulating the expression of the target gene TCF7L2.^42^ In PC, the up‐regulated miR‐802 inhibits the expression of the target gene TCF4, thereby inhibiting the proliferation of tumor cells.^30^ In lung cancer, overexpressed miR‐802 promotes the proliferation of lung cancer cells by targeting the expression of the tumor suppressor gene Menin.^35^ The increased expression of β‐catenin during this process suggests that miR‐802 may activate the Wnt/β‐catenin signaling pathway.^35^

### 4.2 miR‐802 and the PI3K/AKT and ERK signaling pathways

The PI3K/AKT signaling pathway is important to regulate the initial stage of cancer metastasis and is considered a promising target for the treatment of malignant tumors.^43^ As shown in Figures 1B and 2, miR‐802 regulates the expression of ZNF521 in HCC tissues, thus inhibiting the proliferation and colony formation and promoting apoptosis of HCC cells.^24^ The PI3K/AKT signaling pathway can eliminate the inhibitory effect of ZNF521 on HCC cells, which indicates that the tumor‐promoting effect of miR‐802 is mediated by the PI3K/AKT signaling pathway in HCC.^24^ In NSCLC, highly expressed miR‐802 was found to be able to target to inhibit the expression of FGFR1, p‐PI3K, p‐AKT, and p‐mTOR, thereby inhibiting the proliferation, invasion, metastasis, and colony formation of HCC cells, and inducing apoptosis of HCC cells.^34^ This indicates that miR‐802 can inhibit the phosphorylation of PI3K and AKT, thereby inhibiting the activation of the PI3K/AKT signaling pathway.^34^ In addition, in cholesteatoma, the miR‐802/PTEN axis can inhibit the activation of the PI3K/AKT signaling pathway, thereby inducing the proliferation of cholesteatoma cells.^29^

ERK is an extracellular signal‐regulated kinase in the mitogen‐activated protein kinase (MAPK) pathway, which can regulate a variety of cellular behaviors.^44^ In CRC, RAN is a potential target gene of miR‐802. Highly expressed miR‐802 can significantly inhibit the expression of RAN and promote the activation of the ERK signaling pathway and PI3K/AKT signaling pathway, thereby inhibiting the growth, proliferation, invasion, and metastasis of CRC cells, and promoting cell apoptosis.^12^, ^13^

### 4.3 miR‐802 and the Hedgehog signaling pathway

Hedgehog (Hh) signaling pathway is a key regulator of development, cell proliferation, and stem cell maintenance.^45^ RAB23 is an important member of the RAB family, which is involved in the regulation of the material transport of cell membranes and vesicles, thereby affecting cell proliferation and migration.^46^ RAB23 in the cytoplasm can negatively regulate the Hh signaling pathway.^47^ As shown in Figures 1C and 2, in PCa, the expression of miR‐802 and RAB23 is elevated, which promotes the proliferation and migration of PCa cells.^2^

### 4.4 miR‐802 and the EMT signaling pathway

As an evolutionarily conserved biological process, EMT is related to tumorigenesis and can significantly enhance the ability of cancer cells to invade and metastasize.^48^ As shown in Figure 1D, highly expressed miR‐802 in PCa can inhibit the expression of the target gene Flot2, up‐regulate the expression of E‐cadherin, and decrease the expression of N‐cadherin and Vimentin.^33^ This indicates that the miR‐802/Flot2 axis may inhibit the proliferation, invasion, and metastasis of PCa cells by suppressing EMT and enhancing cell apoptosis.^33^ In CC, the high expression of miR‐802 up‐regulates the expression of E‐cadherin and decreases the expression of N‐cadherin, Vimentin, MMP2, and MMP9.^17^ Therefore, the miR‐802/BTF3 axis can inhibit the metastasis, invasion, and cell viability of CC cells by inhibiting EMT.^17^ However, in bladder urothelial carcinoma, the overexpression of miR‐802 inhibits the expression of E‐cadherin and promotes the expression of N‐cadherin and Vimentin.^26^ This indicates that the miR‐802/CYLD axis can promote EMT, thereby promoting the proliferation and metastasis of cancer cells.^26^

### 4.5 The other mechanisms of miR‐802

The biological effects of miR‐802 on tumor cell proliferation, invasion, metastasis, and apoptosis have been confirmed in a number of studies. As shown in Figure 1E, miR‐802 is involved in several cancers including TSCC, OSCC, ESCC, LSCC, GC, CRC, HCC, BC, EOC, CC, and lung cancer.

In TSCC, miR‐802 can target to inhibit the expression of MAP2K4, promote the expression of E‐cadherin, and inhibit the expression of N‐cadherin, Snail, and Vimentin, thereby inhibiting the invasion and viability of cancer cells.^4^ In OSCC, miR‐802 inhibits the expression of the target gene MET, thereby inhibiting the metastasis, invasion, viability, and colony formation of cancer cells.^6^ In ESCC, miR‐802 targets to inhibit the expression of RAB23, thereby inhibiting the proliferation and metastasis of cancer cells.^7^ In LSCC, miR‐802 inhibits the proliferation, colony formation, and apoptosis of LSCC cells by targeting the expression of ARPP19.^8^ In GC, miR‐802 can inhibit the expression of RAB23, MMP‐2, and MMP‐9, thereby inhibiting the proliferation, invasion, metastasis, and colony formation of GC cells, and inducing the apoptosis of GC cells.^9^ In CRC, overexpression of miR‐802 can activate the expression of HSPA6, thereby inhibiting the invasion, metastasis, and colony formation of CRC cells.^42^ miR‐802 inhibits the proliferation, migration, and invasion of CRC cells in vitro and in vivo by inhibiting the expression of the oncogene UBN2, and at the same time enhances the apoptosis of CRC cells.^14^ In HCC, miR‐802 inhibits T‐cell activity by targeting REDDI.^22^ miR‐802 induces the proliferation, metastasis, and cell cycle progression of HCC cells by inhibiting the expression of RUNX3.^49^ In BC, miR‐802 can promote the expression of cyclins A and B by inhibiting the expression of FoxM1 and p27, thereby inhibiting the proliferation of BC cells.^15^ In EOC, miR‐802 inhibits the expression of YWHAZ, thereby inhibiting the proliferation, metastasis, and invasion of cancer cells, and promoting cancer cell apoptosis.^20^ In CC, miR‐802 inhibits the proliferation and colony formation of CC cells by inhibiting the expression of SRSF9.^18^ miR‐802 inhibits the growth and metastasis of CC cells by silencing the expression of MYLIP.^19^ In lung cancer, miR‐802 inhibits the metastasis and invasion of lung cancer cells by silencing the expression of Rnd3.^36^

## 5 CERNAS RELATED TO MIR‐802 EXPRESSION

**FIGURE 3:**
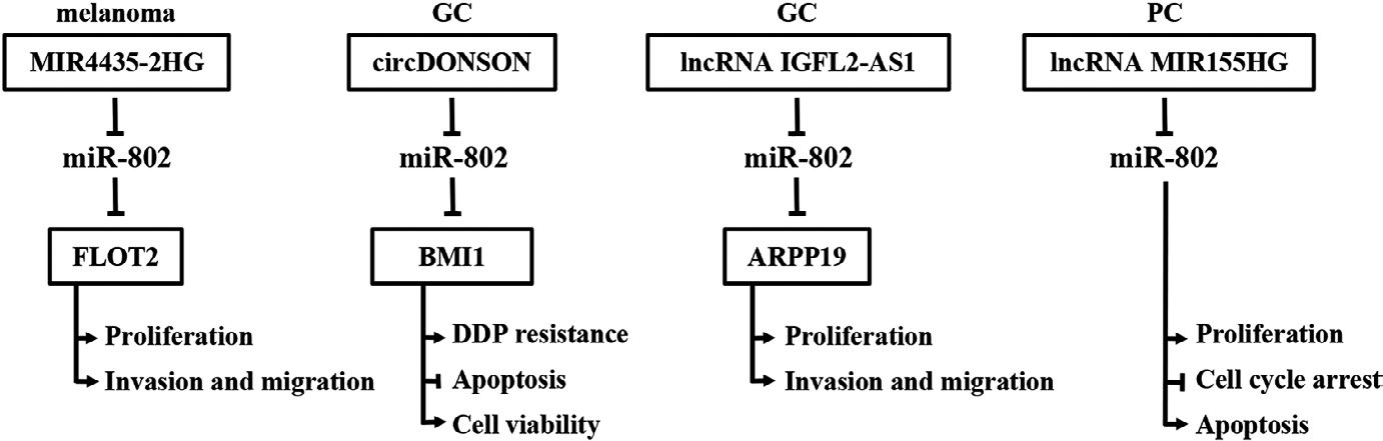
The competing endogenous RNA (ceRNA) mechanisms and potential downstream regulatory mechanisms of miR‐802. In melanoma, GC, and PC, circDONSON, LncRNA IGFL2‐AS1, lncRNA MIR155HG, and lncRNA MIR4435‐2HG can regulate gene expression by competitive binding with miR‐802, forming a ceRNA regulatory network with miR‐802, thereby affecting proliferation, invasion, metastasis, apoptosis, and DDP resistance of tumor cells. ↑, promotion; ↓, inhibition; DDP, cisplatin; GC, gastric cancer; PC, pancreatic cancer

The competing endogenous (ceRNA) can competitively combine with miRNAs to play a transcriptional regulatory role of protein‐coding genes.^50^ As shown in Figure 3, four ceRNAs, including circDONSON, IGFL2‐AS1, MIR155HG, and MIR4435‐2HG, can endogenously compete with miR‐802, thereby affecting tumor progression.

In cisplatin‐resistant GC tissues and cell lines, the expression of circDONSON was significantly increased, and circDONSON could indirectly down‐regulate the expression of BMI1 by competitively binding miR‐802.^10^ Knockout of circDONSON can inhibit the cell viability and promote the production of cisplatin‐resistant cells, and the effect of cell recovery on cisplatin.^10^

In addition, Xu et al.^11^ found that in GC tissues and cell lines, high expression of LncRNA IGFL2‐AS1 inhibits the expression of miR‐802. ARPP19 is a direct target of miR‐802 and can reverse the inhibitory function of miR‐802. It indicates that the IGFL2‐AS1/miR‐802/ARPP19 axis plays a key role in the proliferation, migration, and invasion of GC cells.^11^

Qin et al.^31^ found that the expression of lncRNA MIR155HG in PC tumor tissues and cells is significantly increased. MIR155HG promotes the proliferation of PC cells by sponging miR‐802 and inhibits the apoptosis of PC cells.

The expression of lncRNA MIR4435‐2HG was up‐regulated in melanoma, and lncRNA MIR4435‐2HG could promote proliferation, migration, and invasion of melanoma cells by inhibiting miR‐802 and up‐regulating FLOT2.^21^

## 6 CONCLUSIONS

miR‐802 has biological effects on tumor cell proliferation, metastasis, invasion, and apoptosis. miR‐802 inhibits the expression of numerous target genes, thereby affecting the different mechanisms and signaling pathways of tumorigenesis and development. In addition, this review summarizes the four ceRNAs, circDONSON, LncRNA IGFL2‐AS1, lncRNA MIR155HG, and lncRNA MIR4435‐2HG, which can endogenously compete with miR‐802, thereby affecting tumor progression.

MicroRNA can be used as a high‐quality diagnostic marker of disease. MicroRNA exists in large amounts in serum and plasma, and it can be encapsulated in exosomes, vesicles, and apoptotic bodies to avoid degradation.^51^ This review summarizes significant differences in the expression levels of miR‐802 in several diseases, and these differences can provide a potential theoretical basis for miR‐802 as a diagnostic biomarker of diseases. MicroRNA is a potential therapeutic target, but its off‐target, half‐life, and other issues have always been obstacles to the entry of microRNA into clinical applications.^52^ It is currently known that circDONSON,^10^ lncRNA MIR4435‐2HG,^21^ and lncRNA IGFL2‐AS1^11^ can sponge miR‐802, which can precisely regulate the biological functions of miR‐802. These molecular mechanisms also provide some hints for the future development of miR‐802 in the treatment of related diseases.

There are inconsistent results for the expression of miR‐802 in some tumors. This may be due to the following reasons. Firstly, there are differences in the sample types of different experiments. In the study of pancreatic cancer, miR‐802 was down‐regulated in pancreatic cancer tissues,^30^, ^31^ while it was up‐regulated in the cyst fluid of high‐grade invasive pancreatic cystic lesions.^32^ In addition, miR‐802 was significantly down‐regulated in NSCLC and A549, H522, H1299, H460, and SK‐MES‐1.^34^ Another study found that the expression was up‐regulated in lung cancer tissues, but this study did not specify the subtype of lung cancer.^35^ Secondly, there are differences in the experimental methods for detecting miR‐802 in different studies. The up‐regulation of miR‐802 expression in the cyst fluid of high‐grade invasive pancreatic cystic lesions is based on the RNA‐sequencing results of 4 samples. This finding lacked subsequent RT‐qPCR verification.^32^ Thirdly, miR‐802 can promote and inhibit the same signaling pathway at the same time (Figure 2). In CRC, miR‐802 reduces the activation of the PI3K/AKT/mTOR pathway by down‐regulating RAN, thereby inhibiting tumor cell proliferation, metastasis, and invasion.^13^ In NSCLC, miR‐802 can down‐regulate the expression of FGFR1, thereby inactivating the PI3K/AKT/mTOR pathway and inhibiting the malignant development of NSCLC.^34^ Similarly, in HCC, miR‐802 can promote the PI3K/AKT pathway by inhibiting the expression of ZNF521, and promote cell proliferation and colony formation.^24^ In cholesteatoma, miR‐802 reduces the inhibitory effect of PTEN on the PI3K/AKT pathway by inhibiting the expression of PTEN, thereby promoting the proliferation of tumor cells.^29^ In summary, we believe that the above speculations may explain, at least in part, the inconsistent expressions of miR‐802 in the above‐mentioned tumors.

The current research on miR‐802 still has some limitations. Many studies have initially explored the relevance of miR‐802 and its target genes in cancers, but the specific mechanism is still elusive. For example, in the study of GC, the IGFL2‐AS1/miR‐802/ARPP19 axis needs to be verified in other tumors.^11^ The YWHAZ signaling pathway mediated by miR‐802 may play a role in EOC,^20^ but the specific regulatory mechanism needs to be further studied. MIR155HG promotes tumorigenesis and development through the negative regulation of miR‐802 in PC cells, but whether other mechanisms or targets are involved in the regulation of MIR155HG still needs to be explored.^31^ The mechanism by which miR‐802 regulates pancreatic β‐cell apoptosis still needs to be further studied.^53^ In addition, the experimental samples or models in some studies are very limited. In the study of CC, multiple CC cell lines were not used, and no in vivo experiments were performed.^17^ Research on miR‐802 and HBV lacks tissues and reliable animal samples that are susceptible to HBV infection and lacks in vivo experiments.^23^ In the study of CRC, only the HCT‐116 cell line^12^ was used. In the study of HCC, the overexpression of miR‐802 was only based on SMMU‐7721 cancer cells.^49^ In oral lichen planus (OLP), the effect of miR‐802 on apoptosis has not been validated in OLP animal models.^54^ In order to make the results more convincing, it is necessary to conduct further research on miR‐802 in more comprehensive and diverse samples and models.

This article reviews the aberrant expression of miR‐802 in different tumors can regulate the expression of target genes and the activation or inhibition of different signaling pathways. At the same time, miR‐802 can also serve as a potential molecular indicator for the prognosis of human tumors, providing new ideas for the diagnosis and treatment of tumors in the future.

## CONFLICT OF INTEREST

All authors declare no conflicts of interest.

## AUTHOR CONTRIBUTIONS

TG, MZ, TS, and SD collected and analyzed the related publications, and wrote this study; SD, MZ, and TG conceived and gave the final approval of the submitted version.

## Data Availability

The data presented in this study can be found in online repositories. The name of repositories and reference number can be found in the review.
